# Readiness to provide child health services in rural Uttar Pradesh, India: mapping, monitoring and ongoing supportive supervision

**DOI:** 10.1186/s12913-021-06909-z

**Published:** 2021-09-04

**Authors:** Lorine Pelly, Kanchan Srivastava, Dinesh Singh, Parwez Anis, Vishal Babu Mhadeshwar, Rashmi Kumar, Maryanne Crockett

**Affiliations:** 1 Institute for Global Public Health, University of Manitoba, R070 Med Rehab Building, 771 McDermot Avenue, R3E 0T6 Winnipeg, Manitoba Canada; 2 India Health Action Trust, 404, 4th Floor, No. 20-A Ratan Square, Vidhan Sabha Marg, 226001 Lucknow, Uttar Pradesh India; 3 Department of Pediatrics, King George’s Medical University, King George’s Medical University Chowk, 226003 Lucknow, Uttar Pradesh India; 4 Departments of Pediatrics and Child Health, Medical Microbiology and Infectious Diseases and Community Health Sciences, University of Manitoba, Winnipeg, Canada

**Keywords:** Sustainable development goals, Advocacy, Evaluation, Child health, Facility readiness

## Abstract

**Background:**

In 2018, 875 000 under-five children died in India with children from poor families and rural communities disproportionately affected. Community health centres are positioned to improve access to quality child health services but capacity is often low and the systems for improvements are weak.

**Methods:**

Secondary analysis of child health program data from the Uttar Pradesh Technical Support Unit was used to delineate how program activities were temporally related to public facility readiness to provide child health services including inpatient admissions. Fifteen community health centres were mapped regarding capacity to provide child health services in July 2015. Mapped domains included human resources and training, infrastructure, equipment, drugs/supplies and child health services. Results were disseminated to district health managers. Six months following dissemination, Clinical Support Officers began regular supportive supervision and gaps were discussed monthly with health managers. Senior pediatric residents mentored medical officers over a three-month period. Improvements were assessed using a composite score of facility readiness for child health services in July 2016. Usage of outpatient and inpatient services by under-five children was also assessed.

**Results:**

The median essential composition score increased from 0.59 to 0.78 between July 2015 and July 2016 (maximum score of 1) and the median desirable composite increased from 0.44 to 0.58. The components contributing most to the change were equipment, drugs and supplies and service provision. Scores for trained human resources and infrastructure did not change between assessments. The number of facilities providing some admission services for sick children increased from 1 in July 2015 to 9 in October 2016.

**Conclusions:**

Facility readiness for the provision of child health services in Uttar Pradesh was improved with relatively low inputs and targeted assessment. However, these improvements were only translated into admissions for sick children when clinical mentoring was included in the support provided to facilities.

**Supplementary Information:**

The online version contains supplementary material available at 10.1186/s12913-021-06909-z.

## Background

The National Health Policy of the Government of India (GoI) [1] outlines ambitious plans for the reorganization of the delivery of public health care in India with a goal of providing high quality universal health coverage. Key to this goal is moving from a model of selective primary care to one of available and free comprehensive primary care and strong referral linkages to higher levels of care as required. The policy recognizes the importance of general health systems strengthening to achieve improved outcomes for the national health programs including improving child survival. As part of the policy, public facilities will provide assured free drugs, diagnostics and emergency services. The focus on quality in the policy is especially vital as the achievement of universal health coverage without improved quality will not ultimately change outcomes [2].

The critical relationships for improving quality in health systems are those that exist between policy and strategy development, health service provision and communities and service users [3]. Improving the quality of health service provision at a population level is complex in practice, but in concept it can be distilled down to structures, processes and outcomes [4]. A number of tools and approaches exist for measuring different aspects of service provision quality for health facilities. Some measure only the structural portion of quality improvement such as the World Health Organization’s (WHO) Service Availability and Readiness Survey (SARA) [5]. Others measure multiple dimensions such as the GoI National Quality Assurance Standards (NQAS) [6]. General improvements in all of these aspects of quality at health facilities are beneficial for children for whom care is sought at those facilities. The problem with broad quality assessments, such as SARA and NQAS, is that service readiness for care of sick children can easily become masked as the majority of the data elements and indicators are not specific for pediatric care. These broad-based facility quality assessments are vital for health system improvement but may not specifically assess the availability and readiness of child health services at a facility.

The importance of improving quality of care in low- and middle-income countries is well recognized [7]. Quality of care approaches are more mature for emergency obstetrical care with the use of signal functions to bring focus to the provision of high priority interventions [8]. Building on the research around emergency obstetrical care, assessments of neonatal quality of care have advanced for the immediate post-natal period [9–12]. However, standards for pediatric quality of care have lagged behind other areas with the WHO publishing the first standards in 2018 [13]. A clear understanding of how programs can improve pediatric quality of care and specifically how this can be done within government systems is even less well defined.

Uttar Pradesh (UP), the most populous state in India, has an under-five (U5) child mortality rate of 78 per 1000 live births, one of the highest in the country [14]. One factor contributing to the high U5MR is the high proportion of the population living in rural areas (77.7 %) which may limit access to child health services [15]. In UP, the mortality rate among U5 children living rurally (82 per 1000 live births) is more than 30 % higher than those living in urban areas (62 per 1000 live births) [14]. According to the National Sample Survey 2014 [16], the proportion of U5 children with a reported illness requiring treatment in the 15 days prior to the survey was 103 per 1000 U5 population in rural settings and 114 in urban settings. While there was no gender difference in urban areas, in rural areas the reporting of illness was 119 per 1000 U5 for males and 86 per 1000 U5 for females. The hospitalization rates in the previous year for U5 children were 31 per 1000 U5 population in rural areas (38 for males and 22 for females) and 45 per 1000 U5 population in urban areas (57 for males and 39 for females) [16]. There are higher mortality rates in the rural areas but lower identification of illnesses requiring treatment and lower hospitalization rates. This suggests different illness recognition and care-seeking patterns in rural areas that likely adversely affect the survival of young children.

In general, those in rural settings sought treatment for illness with public providers 28.3 % of the time compared to 21.2 % in urban settings. If hospitalization was required, there were dramatic differences between those who accessed public or private care depending on wealth quintile. Among those who lived rurally, 57.5 % in the poorest wealth quintile were admitted to a public facility compared to 28.9 % in the richest wealth quintile. In urban areas, the results were similar with 48 % in the lowest quintile admitted to public facilities compared to 18.7 % in the richest quintile [16]. Based on the utilization differences between the poorest and richest wealth quintiles, it can be anticipated that programs to scale-up implementation of quality pediatric services in public health facilities in India would preferentially benefit those in the lowest wealth quintiles and those who live in rural areas.

### Program Context

Since 2014, the University of Manitoba and India Health Action Trust have been supporting the Government of Uttar Pradesh (GoUP) through the Uttar Pradesh Technical Support Unit (UP-TSU) for Reproductive, Maternal, Newborn, Child and Adolescent Health and Nutrition which provides techno-managerial support at the state-, district- and block-level in 25 high priority districts (HPDs) in UP. The Child Health program of the UP-TSU was launched in November 2014 as a learning project to understand how interventions to reduce morbidity and mortality in children with pneumonia and diarrhoea could be implemented in UP in a manner that would be sustainable through government systems. The Department of Pediatrics at King George’s Medical University (KGMU) was a partner in the project.

UP is comprised of 75 districts and 820 blocks. Public health facilities in India are structured to include sub-centres, primary health centres, community health centres (CHC), sub-district hospitals, district hospitals and medical colleges [17]. CHCs are the highest facility at the block level. According to the Indian Public Health Standards (IPHS) updated in 2012 [18], a CHC should provide ‘routine and emergency care for sick children including facility-based integrated management of neonatal and childhood illnesses (F-IMNCI) strategy’ (p.4) as an essential service. The minimum human resources suggested in these national guidelines to provide essential services in surgery, maternal health, newborn and child health, family planning and other national health programs, includes five specialists (including one pediatrician), two allopathic medical officers (MOs), ten staff nurses, one pharmacist, two lab technicians and one X-ray technician. The CHCs are well-positioned to provide care for children in rural communities that bear the highest rates of child deaths.

The aim of this paper is to use secondary analysis of program data to delineate how activities in the programs were temporally-related to readiness of public facilities to provide child health services including inpatient admissions. This analysis was conducted to understand the effects of the program activities and the process and potential use of specific facility readiness assessments in the context of strengthening child health services in UP.

## Methods

 The underlying theory of change for the intervention was that key inputs required to provide care for sick children needed to be present and functional in facilities to improve the quality of care and ultimately decrease morbidity and mortality. These inputs have been delineated in national-level guidelines but implementation has remained a challenge. Among the key inputs, some are largely within the control of the facility and district health officials and improvements can be made relatively quickly with sustained focus. However, some inputs require higher level intervention and capital costs such as human resources and many aspects of infrastructure which move more slowly.

Three initial intervention districts were purposively selected from the 25 HPDs covered by the UP-TSU to exploit maximum geographic variability. Prior to initiating program activities, 15 CHCs in the intervention districts were mapped to define the gaps in readiness to deliver quality child health care at these facilities from May to July 2015. The child health facility mapping tools were based on previous facility mapping tools developed by the UP-TSU but were adjusted to reflect operational guidelines from the GoI and National Health Mission (NHM) on F-IMNCI [19] and IPHS for CHCs [18]. Mapped domains included human resources and training, infrastructure, equipment, drugs and supplies and reported provision of child health services.

Following the facility mapping exercise ending in July 2015, facility- and district-specific reports were prepared and the results were disseminated to facility and district health managers in each of the three intervention districts. The output of the dissemination meetings was facility-specific plans for improvement that were developed in concert with the involved health officials. The program hired staff at the district level who were directly interacting with district health officials and facility staff at each intervention facility to follow up on the recommendations for facility improvement that had been agreed upon in the dissemination meetings.

Beginning in November 2015, clinical support officers (CSOs), hired through KGMU, began monthly monitoring of the intervention facilities and provided regular supportive supervision to the staff. Monthly monitoring of facility U5 service statistics began in March 2016. There was a delay in collection of service statistics as there was an initial need to improve record keeping. One of the interventions to improve record keeping was the use of a daily stamp to summarize the number of U5 children assessed in the outpatient department, disaggregated by acute flaccid paralysis, measles, pneumonia and diarrhoea. The intention had been to hire staff with allopathic medical degrees for these positions but it was not possible to recruit staff with this training under the maximum salary structure available for non-tenured positions through a government funded university. Accordingly, physicians trained in traditional Indian medical systems with experience working in allopathic clinical settings were hired. A formal program to develop competency in key pediatric skills for the CSOs was completed under the supervision of pediatricians at KGMU. Additionally, from April to June 2016, senior pediatric residents from KGMU were placed with the project as an elective rotation within their pediatric residency program. The senior pediatric residents provided on-site mentoring to facility staff at all of the intervention facilities. There was restructuring in the Pediatric Department at KGMU in July 2016 and the elective option was discontinued. Additionally, 12 of the 15 facilities had the continuous presence of a Nurse Mentor who worked as part of the broader programs of the UP-TSU and supported the facility staff in child health initiatives between visits of the other team members.

**Fig. 1:**
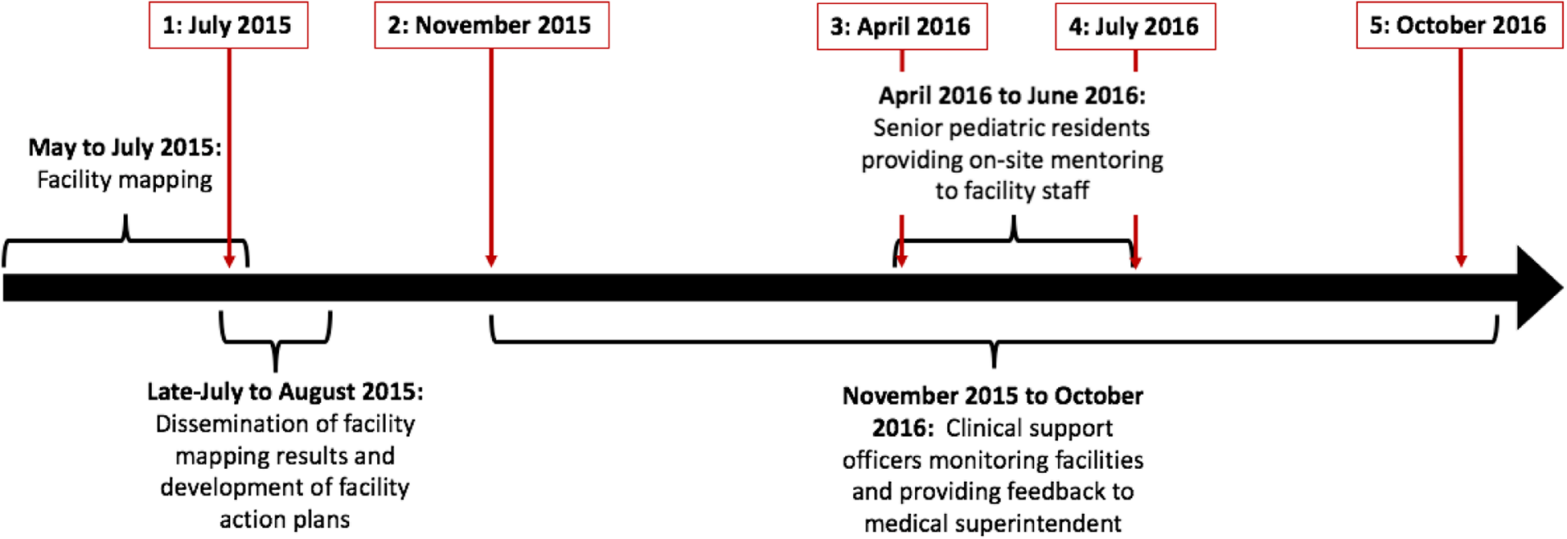
Timeline of program interventions at 15 CHCs in UP with five key time points

Concurrently, specific plans were also developed with the GoUP and NHM officials to address the barriers to timely, high quality child health services. Due to programmatic changes and integration with other UP-TSU programs, the role of the CSOs was discontinued after October 2016. Key time points in the program are shown in Fig. 1.

### Data Sources for the Facility Readiness Assessment

The mapped elements included infrastructure, human resources, drugs and supplies, equipment and reported service provision. Infrastructure, drugs and supplies and equipment elements were directly observed. Infrastructure elements received a score of one if they were available and, if relevant, functional. Drugs and supplies needed to be available, unexpired and/or functional to receive a score of one. Equipment needed to be available and functional to receive a score of one. Human resources and service provision were reported data elements with information obtained from the medical officer-in-charge. The reported service provision data elements were considered a measure of facility willingness to provide these services and received a score of one if the service was reported as being provided. Initial data on the readiness of facilities for the provision of child health services was collected by the program team during the facility mapping exercise that took place from May to July 2015. From November 2015 to October 2016, monthly data was collected on drugs and supplies and equipment availability by the CSOs. In July 2016, data on infrastructure, human resources and reported service provision was recollected by the program team.

### Data Sources for U5 Patient Volumes

Data on the number of U5 children seen in the outpatient department (OPD) and the inpatient department (IPD) were collected monthly from March to October 2016 by the CSOs from the OPD and IPD registers at each facility.

### Analysis

The facility mapping and program monitoring data was analyzed through the lens of five important time points in the delivery of the program (Fig. 1). The full sets of facility readiness data from July 2015 to July 2016 were compared using essential and desirable composite scores and component scores for facility readiness to provide child health services at CHCs. The structure of the composite score that was used is available in the supplementary material. To summarize, essential and desirable composite scores for readiness to provide child health services at CHCs were calculated based on criteria for strengthening that had been developed previously with government counterparts using government guidelines. The maximum score for the essential and desirable composite scores was one with each of the five components – infrastructure, human resources, drugs and supplies, equipment and reported service provision – contributing to the composite score in equal parts. If a component score had sub-components, then each of the sub-components contributed to the component score in equal parts.

Non-parametric measures were used to compare median essential and desirable composite scores using Stata version 15 (StataCorp LLC). Component scores for drugs and supplies and equipment were calculated at five key time points using Microsoft Excel (Version 15.0).

### Ethics and Approvals

The overall project received approval through the institutional review boards of the University of Manitoba and KGMU. Approval for secondary data analysis of program data for this paper was given by the University of Manitoba. Additionally, the GoUP issued a government order approving this project.

## Results

### Overall facility strengthening

**Fig. 2:**
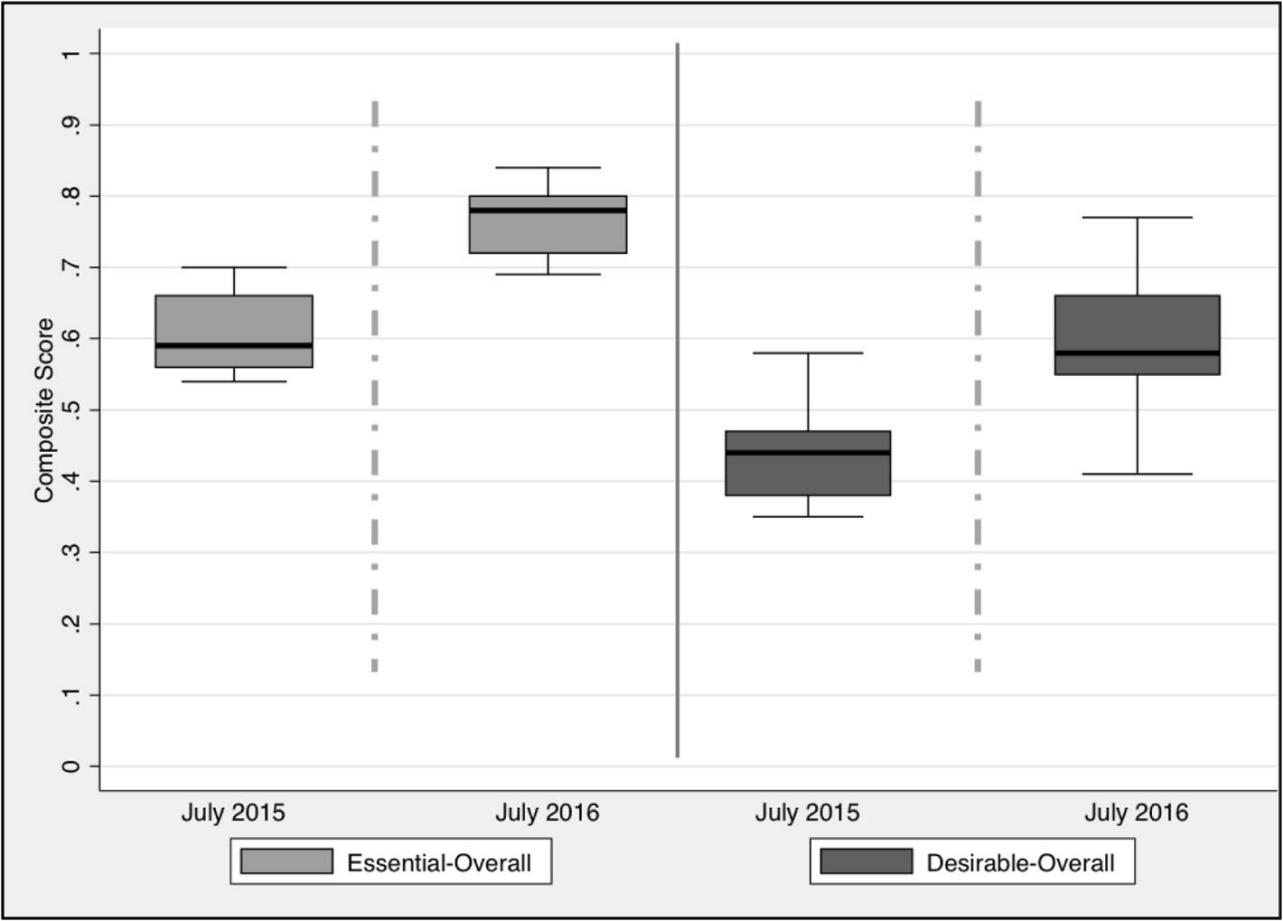
Essential and desirable composite scores between July 2015 and July 2016

Comparing the overall composite score between July 2015 and July 2016, the median essential composite score of the 15 facilities increased from 0.59 to 0.78 (*p* < 0.0001) and the median desirable composite score increased from 0.44 to 0.58 (*p* < 0.0001) (Table 1; Fig. 2). The majority of the improvement in both essential and desirable composites scores between July 2015 and July 2016 was accounted for by increases in the drugs and supplies, equipment and service provision components (Table 1). As shown in Fig. 2, the data clustered more tightly around the median in the essential composite score than the desirable composite score indicating greater heterogeneity for the desirable composite score among facilities. Detailed data sets as well as component and composite scoring are available in the supplementary material.

**Table 1:** Median essential and desirable composite scores (with ranges) for facility readiness of child health services in intervention CHCs (*n* = 15) between July 2015 and July 2016

	July 2015	July 2016	Change
**Essential Score**	**0.59 (0.54 to 0.7)**	**0.78 (0.69 to 0.84)**	+ 0.19 (*p* < 0.001*)
Drugs and Supplies	0.52 (0.39 to 0.77)	0.73 (0.55 to 0.91)	+ 0.21
Equipment	0.25 (0.15 to 0.52)	0.73 (0.43 to 0.83)	+ 0.48
Trained HR	0.6 (0.4 to 0.6)	0.6 (04 to 0.8)	0
Infrastructure	1 (0.8 to 1)	1 (1 to 1)	0
Service Provision	0.68 (0.53 to 0.85)	0.82 (0.6 to 0.88)	+ 0.14
**Desirable Score**	**0.44 (0.35 to 0.58)**	**0.58 (0.41 to 0.77)**	+ 0.14 (*p* < 0.001*)
Drugs and Supplies	0.44 (0.33 to 0.69)	0.69 (0.47 to 0.9)	+ 0.25
Equipment	0.14 (0.06 to 0.31)	0.57 (0.24 to 0.67)	+ 0.43
Trained HR	0.43 (0.14 to 0.71)	0.43 (0.14 to 0.71)	0
Infrastructure	0.6 (0.4 to 0.8)	0.6 (0.5 to 0.91)	0
Service Provision	0.6 (0.45 to 0.75)	0.78 (0.37 to 0.83)	+ 0.18

* Used the Kruskal-Wallis equality-of-populations rank test (essential score - chi-squared 21.197 with 1 d.f.; desirable score - chi-squared 16.188 with 1 d.f.)

### Infrastructure

At baseline, all 15 facilities already had at least four of the five essential infrastructure components including computer with internet, lab facility, ambulance facility and pharmacy. In 2015, only seven of the facilities had space allocated for emergency care (either a separate room or a dedicated space) which could be used 24 h a day indicating there was grid-attached power to the space with back-up from either a generator or inverter. By 2016, an emergency space had been allocated in all 15 facilities. In terms of desirable infrastructure, no significant improvement was achieved.

### Human Resources

Improvement in either essential or desirable human resources (HR) component score was not seen between July 2015 and July 2016. However, in 2015, none of the facilities had an MO trained in F-IMNCI and in 2016, six facilities had at least one MO trained in F-IMNCI.

### Drugs and Supplies

**Fig. 3:**
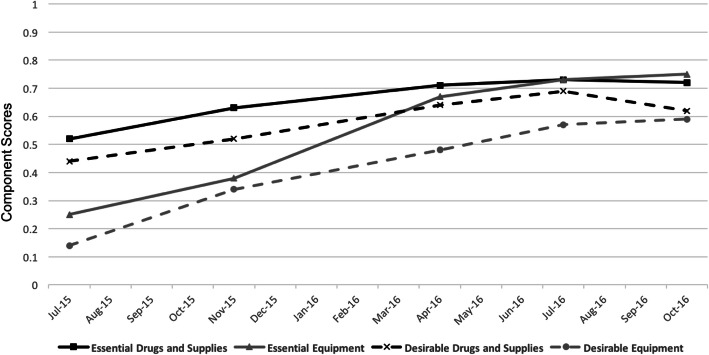
Median component score for drugs and supplies and equipment over five key project time periods

The essential and desirable drugs and supplies component scores increased from 0.52 to 0.73 and 0.44 to 0.69, respectively, between July 2015 and July 2016 (Table 1; Fig. 3). The subcomponents that accounted for most of the increases seen were drugs and supplies for the emergency kits. The subcomponent scores increased from 0.67 to 0.83 for essential emergency drugs and from 0.6 to 0.8 for essential emergency supplies. Unfortunately, the drugs and supplies component scores decreased slightly between July 2016 and October 2016 (Fig. 3) largely due to a decrease in the availability of ‘other child health drugs’ such as third generation cephalosporins, salbutamol nebulizing solution, ampicillin and paracetamol.

### Equipment

The largest improvements that occurred were seen in the essential and desirable equipment components (Table 1; Fig. 3). The essential equipment component, composed of equipment in the OPD and the emergency area, increased from 0.25 to 0.73. The desirable equipment score, composed of equipment in the OPD, IPD, emergency and newborn stabilization unit (NBSU) increased from 0.14 to 0.57. In particular, the IPD equipment subcomponent score in the desirable composite score increased from 0.09 to 0.91 (supplemental material). NBSU equipment was included in the desirable composite score so that the results would be comparable to future data but the data elements to compute this subcomponent were not available in either July 2015 or 2016.

### Service Provision

Improvement was also seen in the reported provision of regular child health services with the essential component score increasing from 0.68 to 0.82 and the desirable component score increasing from 0.6 to 0.78 (Table 1). While essential newborn service provision was unchanged, there were increases in the reported OPD and emergency services provided regularly to U5 children.

### Volume of Under-Five Children in Outpatient and Inpatient Departments

Among the 15 intervention facilities, 3823 to 8658 U5 children were seen in the OPD every month (average of 255 to 577 U5 children per facility per month). Between 15 and 22 % of the cases seen each month were recorded as being either pneumonia or diarrhoea, respectively. Inpatient admissions increased between March and October 2016 with 0.04 % of U5 OPD assessments being converted to admissions in March and 1.52 % being converted in October. The majority of the inpatient admissions in April to July were diarrhoea cases with a mostly equal balance of pneumonia and diarrhoea cases admitted in September and October.

**Fig. 4:**
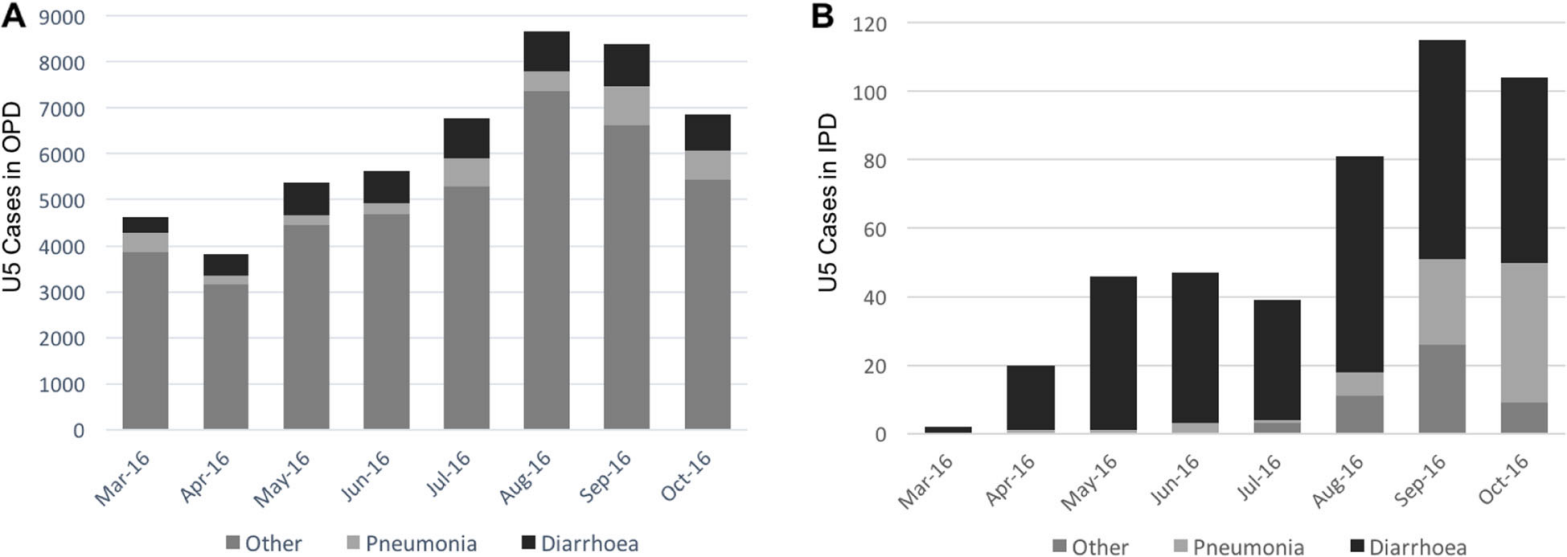
Child health service statistics for (**A**) outpatient departments and (**B**) inpatient departments from March 2016 to October 2016

## Discussion

Achieving adequate facility readiness to provide specific child health services is a complex process. It requires trained HR who can provide the necessary services in an environment with adequate infrastructure, drugs and supplies and equipment. Facility readiness on its own is insufficient for providing high quality services [20]; however, it is an important first step.

Strengthening health services in low- and middle-income countries is challenging and the evidence base is insufficient to support any specific strategy as applicable across all geographies [21]. It was noted that the ability to adapt a strategy to the local context led to better implementation and addressing barriers and constraints with stakeholders at the local level improved sucesss. Accessibility of adequate resources was associated with successful implementation of strategies although it was not sufficient on its own.

Broader health system strengthening programs have been associated with improved district facility readiness [22]. In Rwanda, the DHS Program Service Provision Assessment (SPA) tool, in which sick child care is included, was used for an initial gap assessment and to facilitate data-driven improvements. This intervention utilized government systems and improvements were seen in multiple domains including infrastructure, data use, clinicals services and medical equipment and were sustained for two years after intensive support was provided. Recently published evaluations looking specifically at pediatric quality of care in facilities in low-and middle income countries use data from the World Bank Service Delivery Indicators or an expanded version of the Service Provision Assessment; however, they are cross-sectional and focus only on the outpatient clinical areas [23, 24]. In one study, 42 % of facilities were stocked with at least one type of medication relevant to four service areas and only 70 % had all three pieces of essential medical equipment [23]. Cross-sectional assessments are vital as they identify what domains need strengthening and look at potential associations among different aspects of quality of care. In India, a cross-sectional maternal and newborn facility readiness assessment was completed in Bihar and found that approximately half of the essential drugs and about 70 % of the essential equipment were available [25]. In other sites in South Asia implementing programs that included strengthening different aspects of facility-based neonatal care, important changes in clinical service were observed but either drugs and supplies were externally supplemented [26] or improvements in drugs, supplies and equipment were limited [27].

In improving health service delivery, the question of ‘how” something can work is central [21] and contextual. To that end, the goal of this discussion is to carefully reflect on the details of the implementation strategy including the adjustments that were made during the program as we learned more about what was and was not working.

Following the initial facility mapping and dissemination of results to each facility and the district health managers in 2015, the primary method of catalyzing improvements was through a program person placed in each district with post-graduate education in health management. Those without previous experience in public health received additional mentoring early in the project. These district program personnel worked very closely with the district government officials, such as the chief medical officers (CMOs) and the additional CMOs, and supported the CMO’s office in the District Health Society meetings chaired by the District Magistrate. They also supported the medical officers in charge (MOICs) at each of the facilities. The addition of this person who focused on strengthening services for child health led to improvements in the drugs and supplies and equipment component scores for both the essential and desirable composite scores (Fig. 3).

When the Clinical Support Officers (CSOs) started in November 2015, there were continued improvements in the drugs and supplies and equipment component scores with accelerated improvement in essential equipment. The CSOs worked closely with the staff at the facilities to move equipment from store rooms out into the clinical areas where they were required and to work with other UP-TSU district-level program staff to move equipment from the district supply depots to the facilities. The CSOs advocated for MOs from the facilities to be trained in F-IMNCI, the government child health package. Although the CSOs were able to advocate for training and mobilize resources, because they were not credentialed within the allopathic system, they did not have the standing to mentor the MOs and catalyze admissions to the CHC inpatient areas.

U5 IPD admissions accelerated during the period of time that three senior pediatric residents were on an elective rotation with the program and were visiting each facility for at least two days every month (Fig. 4B). During these visits, they mentored the MOs at the facility on management and ongoing monitoring of children with severe dehydration, use of oxygen and inpatient management of pneumonia and other technical skills such as IV starts, NG insertion and using pulse oximeters for monitoring oxygen requirements. These skill development activities had been planned to be supported by the CSOs who had been specifically trained on these skills at KGMU; however, the MOs were not receptive to clinical skill mentoring from non-allopathic physicians, despite their technical capacity in these skills.

As seen in Fig. 4, the initial increase in admissions was primarily for diarrhoea cases in part due to seasonality. The facility staff generally felt that diarrhoea was easier to manage at the CHC level and this belief was leveraged to help MOs gain confidence in admitting sick children to the IPD. Once a facility was admitting regularly, even if only for diarrhoea cases, there was opportunity to support admissions for other illnesses such as pneumonia. The addition of the senior pediatric residents to the program team was temporally related to an increase in admissions for non-diarrhoeal illnesses. The most severe cases continued to be referred to higher facilities. In July 2015, only one facility was routinely providing at least some admission services to U5 children; however, this increased to nine of the fifteen facilities by October 2016.

Managerial interventions such as supervision have been shown to be effective in low-resource settings [28]. In the case of supportive supervision, often the same term is used to include a variety of different tasks and roles [29]. We used a multi-layered supportive supervision approach acknowledging that supporting clinical services may require different people in different roles. The impact of the supervision also depends on the perceived skill and ability of the supervisor [30]. The relationship between coaching-based interventions and outcomes is complex and depends on a broader context. An example of this is the implementation of the Better Birth Checklist in Uttar Pradesh that did not show a relationship between improved practices and decreased mortality [31]. In other settings and conditions, supportive supervision has been shown to be helpful, however, the context and details are important [32–34].

During the analyzed time period, we did not see significant improvements in human resources or infrastructure. This is not unexpected given that changes in infrastructure and staffing require capital expenditures and higher level policy changes requiring a longer timeframe. Supported by the Health Systems team of the UP-TSU, the GoUP is making important changes to meet human resource needs including hiring an additional 10,000 staff nurses to meet IPHS standards for CHCs and district hospitals.

Throughout the program, the facility readiness assessment results were fed back immediately to the facility with specific supports provided to initiate the required improvements. The benefit of using the composite scoring has been the ability to identify how the system is moving, and the ability to be able to understand at an aggregate level which components contain the critical systems issues, while also being able to drill down at a granular level to which component was an issue in an individual facility and to understand within that component exactly what was creating the deficit. Some actions to rectify the identified problems could then be taken at the facility or district level while other problems needed to advance to the divisional, state and even national level.

This program highlighted that facility-specific data needs to be available and used by the facility staff to improve facility readiness to provide child health services. With focused attention and knowledgeable support, progress can be made to improve structural facility readiness. However, this is not likely to translate into the provision of clinical services for children without specific support from individuals with status within the allopathic medical system.

In terms of sustainability and generalizability, the strength of this intervention was that improvements occurred through government procurement processes and systems. Following the work in three districts as outlined in this manuscript, the intervention was adapted and scaled up to 25 district hospitals and 100 CHCs in the 25 high-priority districts covered by the UP-TSU. Additionally, the Government of UP has put in place a number of programs and systems including for child health that will support ongoing facility readiness. These include scaling up the Nurse Mentoring program to all blocks in the state [35], supporting physicians providing RMNCH services through Regional Resource Training Centres [36, 37], continuing to invest in and scale-up the work of the Quality Assurance Program of the Uttar Pradesh National Health Mission [38] and the operation of the recently developed Uttar Pradesh Medical Supplies Corporation [39].

One of the limitations of this analysis is that while we describe the approach to supporting improvements in the facilities, we have not rigorously evaluated other possible explanations for why the improvements may have occurred. Decision making at the facility level can be very complex and this was not explored in the first phase. Another limitation is that the facility readiness assessments did not use the government data systems so there will be issues with sustainable measurement of facility readiness unless this is rectified.

## Conclusions

 This analysis demonstrates that improvement in facility readiness for provision of child health services could be improved in UP with relatively few inputs. With a targeted assessment and development of action plans including regular follow-up with district health officials, drugs and supplies and equipment availability was substantially improved. This improvement was supported by regular follow-up with facility leadership and embedded facility-level support. However, translating facility readiness into clinical assessments and admissions for sick children only occurred once accepted clinical mentoring was included in the facility support.

This study is relevant because the current challenges in child survival are less about what specific clinical interventions work but rather how they can be implemented in a large and complex context like UP in a manner that is sustainable by the government

## Supplementary Information

**Additional file 1.**

**Additional file 2.**

**Additional file 3.**

## Data Availability

The de-identified datasets generated and/or analysed during the current study are publicly available as an appendix to the paper. For additional information or data regarding the study, please contact the corresponding author.

## Abbreviations

ANM: Auxiliary Nurse Midwife
ASHA: Accredited Social Health Activist
BMGF: Bill and Melinda Gates Foundation
CHC: Community health centre
CMO: Chief medical officer
CSO: Clinical support officer
F-IMNCI: Facility-Based Integrated Management of Neonatal and Childhood Illness
GoI: Government of India
GoUP: Government of Uttar Pradesh
HPD: High-priority districts
HR: Human resources
IPD: Inpatient department
IPHS: Indian Public Health Standards
KGMU: King George’s Medical University
MO: Medical officer
MOIC: Medical officer in charge
NBSU: Newborn stabilization unit
NHM: National Health Mission
NQAS: National Quality Assurance Standards
OPD: Outpatient department
SARA: Service Availability and Readiness Assessment
U5: Under-five
UP: Uttar Pradesh
UP-TSU: Uttar Pradesh Technical Support Unit
WHO: World Health Organization
