# Supplementary material for: Readiness to provide child health services in rural Uttar Pradesh, India: mapping, monitoring and ongoing supportive supervision
Source: BMC Health Serv Res. 2021 Sep 4;21:914. doi: 10.1186/s12913-021-06909-z (PMC8417968; doi:10.1186/s12913-021-06909-z)
Supplement: Supplementary file 1 — Additional file 1. [file 12913_2021_6909_MOESM1_ESM.docx]

*Supplementary Material for* **Readiness to Provide Child Health Services in Rural Uttar Pradesh, India: Mapping, Monitoring and Ongoing Supportive Supervision**

Lorine Pelly*^1^, Kanchan Srivastava^2^, Dinesh Singh^2^, Parwez Anis^2^, Vishal Babu Mhadeshwar^2^, Rashmi Kumar^3^, Maryanne Crockett^1^

^1^University of Manitoba, Institute for Global Public Health, R070 Med Rehab Building, 771 McDermot Avenue, Winnipeg, Manitoba R3E 0T6, Canada

^2^India Health Action Trust, 404, 4^th^ Floor, No. 20-A Ratan Square, Vidhan Sabha Marg, Lucknow, Uttar Pradesh 226001, India

^3^King George’s Medical University, Department of Pediatrics, King George’s Medical University Chowk, Lucknow, Uttar Pradesh 226003, India

* Corresponding author

**Author Information**

Dr. Lorine Pelly

[Lorine.pelly@umanitoba.ca](mailto:Lorine.pelly@umanitoba.ca)

Dr. Kanchan Srivastava

[kanchan.srivastava29@gmail.com](mailto:kanchan.srivastava29@gmail.com)

Dr. Dinesh Singh

[dinesh.singh@ihat.in](mailto:dinesh.singh@ihat.in)

Parwez Anis

[parwezanis@gmail.com](mailto:parwezanis@gmail.com)

Vishal Babu Mhadeshwar

[mhadeshwar16@gmail.com](mailto:mhadeshwar16@gmail.com)

Dr. Rashmi Kumar

[rashmik2005@gmail.com](mailto:rashmik2005@gmail.com)

Dr. Maryanne Crockett

[Maryanne.crockett@umanitoba.ca](mailto:Maryanne.crockett@umanitoba.ca)

Supplemental Table 1. Components of the Essential Composite Score for Facility Readiness at the Community Health Centre Level

|  | Component | Subcomponent | Proportion of composite score | Notes |
| --- | --- | --- | --- | --- |
| Essential Composite Score | | |  | Maximum composite score is 1. Each component is weighted equally. |
|  | Drugs and Supplies | | 0.2 | Each subcomponent is weighted equally. |
|  |  | Pneumonia and Diarrhoea (P&D) Drugs (5 items) | 0.05 | Each item is weighted equally. |
|  |  | Other Child Health Drugs (8 items) | 0.05 |  |
|  |  | Drugs for Emergency Drug Kit (6 items) | 0.05 |  |
|  |  | Supplies for Emergency Equipment Kit (10 items) | 0.05 |  |
|  | Equipment | | 0.2 | Each subcomponent is weighted equally. |
|  |  | Outpatient Department (OPD) (10 items) | 0.1 | Each item is weighted equally. |
|  |  | Emergency Room/Area (ER) (15 items) | 0.1 |  |
|  | Infrastructure (5 items) | | 0.2 | Each item is weighted equally. |
|  | Human Resources (5 items) | | 0.2 | Each item is weighted equally. |
|  | Services | | 0.2 | Each subcomponent is weighted equally. |
|  |  | OPD Services (9 items) | 0.33 | Each item is weighted equally. |
|  |  | ER Services (10 items) | 0.33 |  |
|  |  | Neonatal Services (4 items) | 0.33 |  |

Supplemental Table 2. Components of the Desirable Composite Score for Facility Readiness at the Community Health Centre Level

|  | Component | Subcomponent | Proportion of composite score | Notes |
| --- | --- | --- | --- | --- |
| Desirable Composite Score | | |  | Maximum composite score is 1. Each component is weighted equally. |
|  | Drugs and Supplies | | 0.2 | Each subcomponent is weighted equally. |
|  |  | Pneumonia and Diarrhoea (P&D) Drugs (5 items) | 0.05 | Each item is weighted equally. |
|  |  | Other Child Health Drugs (12 items) | 0.05 |  |
|  |  | Drugs for Emergency Drug Kit (12 items) | 0.05 |  |
|  |  | Supplies for Emergency Equipment Kit (12 items) | 0.05 |  |
|  | Equipment | | 0.2 | Each subcomponent is weighted equally. |
|  |  | Outpatient Department (OPD) (12 items) | 0.05 | Each item is weighted equally. |
|  |  | Inpatient Department (IPD) (11 items) | 0.05 |  |
|  |  | Emergency Room/Area (ER) (19 items) | 0.05 |  |
|  |  | Newborn Stabilization Unit (13 items) | 0.05 |  |
|  | Infrastructure (11 items) | | 0.2 | Each item is weighted equally. |
|  | Human Resources (7 items) | | 0.2 | Each item is weighted equally. |
|  | Services | | 0.2 | Each subcomponent is weighted equally. |
|  |  | OPD Services (11 items) | 0.05 | Each item is weighted equally. |
|  |  | ER Services (12 items) | 0.05 |  |
|  |  | IPD Services (2 items) | 0.05 |  |
|  |  | Neonatal Services (9 items) | 0.05 |  |
